# 

**Published:** 1917-12-22

**Authors:** 


### CONTENTS.

PAGE

**Should the Doctor Tell?** ... ... ... ... 239

**Personal Service Triumphant** ... ... ... 240

**Hospital and Institutional News** ... ... ... 241

King Edward's Hospital Fund—This Week's Illustrations—Dr. Garrett Anderson—The Case of Baguley Sanatorium—The Sir Edward Wood Memorial—Mr. H. H. Wills—The Manchester Radium Committee—What is the Virtue of an Honorary Secretary?—The Truth about German Hospitals—Mothers' Pensions and Juvenile Crime—What Can be Done in a Year!—Promotion in the I.M.S.—Clerk to the Governors of Guy's Hospital—Red Cross Work after the War—Llanelly and Infant Welfare—A Grant for Play Centres—A Monster *Thistle*—The *British Prisoner of War*—No Beds for Diphtheria Patients—This Week's Drug Market.

**Village Practice.—II.** ... ... ... ... ... ... 245

An Autobiographical Fragment.

**The Training of Recruits for Nursing Orderlies** ... ... ... ... ... ... ... ... 247

**Distribution of Medals in an Auxiliary Hospital** ... ... ... ... ... ... 248

PAGE

**King Edward's Hospital Fund for London** ... 249

The Distribution Meeting.—£190,000 Awarded.
Report of the Distribution Committee.
Grants to Hospitals Recommended by the Distribution Committee.
Report of the Convalescent Homes Committee.
Grants Recommended by the Convalescent Homes Committee.

**The Editor's Letter-Box** ... ... ... ... 255

An Idle Rumour Resting on Malice.
Prison for Boys—and the Remedy.

**Parliament and the Profession** ... ... ... 255

**Nursing Affairs in Ireland** ... ... ... ... 256

**The Matrons' and Sisters' Department** ... 257

Nursing Progress and its Developments.
Round the Hospitals.
Challenging the Christmas Spirit—Evidences of Bolo's Defeat—Miss Sidney Browne Joins College Council—Princess Christian and Her Nurses—Nursing Tarradilloes and a Lapse—Christmas in Birmingham.

**Some Hospital Types of British Nursing** ... 259

III. The Medical Ward Sister: "A Sermon on the Mount."

**Matrons' and Sisters' Appointments** ... ... 260

